# Supplementary material for: ProteinShader: illustrative rendering of macromolecules
Source: BMC Struct Biol. 2009 Mar 30;9:19. doi: 10.1186/1472-6807-9-19 (PMC2672931; doi:10.1186/1472-6807-9-19)
Supplement: Additional file 1 — ProteinShader program without source code. This compressed file contains the complete ProteinShader program including associated libraries, but no source code. A README.txt file gives an overview of the ProteinShader distribution, and the index.html file in the help subdirectory has directions on getting started with the program as well as a set of tutorials. [file 1472-6807-9-19-S1.zip › ProteinShader-beta-0_9_4-binary/help/api/org/proteinshader/structure/enums/DecorationEnum.html]

DecorationEnum (ProteinShader API)


|  |  |  |
| --- | --- | --- |
| **Overview** \| **Package** \| **Class** \| **Use** \| **Tree** \| **Deprecated** \| **Index** \| **Help** | |  |
| **PREV CLASS**   **NEXT CLASS** | **FRAMES**    **NO FRAMES**     **All Classes** |  |
| SUMMARY: NESTED \| ENUM CONSTANTS \| FIELD \| METHOD | DETAIL: ENUM CONSTANTS \| FIELD \| METHOD |  |


---


## org.proteinshader.structure.enums Enum DecorationEnum

```
java.lang.Object
  java.lang.Enum<DecorationEnum>
      org.proteinshader.structure.enums.DecorationEnum
```

**All Implemented Interfaces:**: Serializable, Comparable<DecorationEnum>

---

``` public enum DecorationEnum extends Enum<DecorationEnum> ```

Provides an enumeration to specify if a Segment object should be plain,
with text labels, or with halftoning when the Segment is rendered as a
three-dimensional tube or ribbon.

---

**Enum Constant Summary**

`HALFTONING`

`PATTERNS`

`PLAIN`

`TEXT_LABELS`

`WIREFRAME`


| **Method Summary** | |
| --- | --- |
| `static DecorationEnum` | `valueOf(String name)`             Returns the enum constant of this type with the specified name. |
| `static DecorationEnum[]` | `values()`             Returns an array containing the constants of this enum type, in the order they're declared. |

**Methods inherited from class java.lang.Enum**

`clone, compareTo, equals, getDeclaringClass, hashCode, name, ordinal, toString, valueOf`

**Methods inherited from class java.lang.Object**

`finalize, getClass, notify, notifyAll, wait, wait, wait`

**Enum Constant Detail**

### PLAIN

```
public static final DecorationEnum PLAIN
```

---


### WIREFRAME

```
public static final DecorationEnum WIREFRAME
```

---


### TEXT\_LABELS

```
public static final DecorationEnum TEXT_LABELS
```

---


### PATTERNS

```
public static final DecorationEnum PATTERNS
```

---


### HALFTONING

```
public static final DecorationEnum HALFTONING
```


**Method Detail**

### values

```
public static final DecorationEnum[] values()
```

:   Returns an array containing the constants of this enum type, in
    the order they're declared. This method may be used to iterate
    over the constants as follows:

    ```
    for(DecorationEnum c : DecorationEnum.values())
            System.out.println(c);
    ```

    :   **Returns:**: an array containing the constants of this enum type, in the order they're declared

---


### valueOf

```
public static DecorationEnum valueOf(String name)
```

:   Returns the enum constant of this type with the specified name.
    The string must match *exactly* an identifier used to declare an
    enum constant in this type. (Extraneous whitespace characters are
    not permitted.)

    :   **Parameters:**: `name` - the name of the enum constant to be returned. **Returns:**: the enum constant with the specified name **Throws:**: `IllegalArgumentException` - if this enum type has no constant with the specified name


---


|  |  |  |
| --- | --- | --- |
| **Overview** \| **Package** \| **Class** \| **Use** \| **Tree** \| **Deprecated** \| **Index** \| **Help** | |  |
| **PREV CLASS**   **NEXT CLASS** | **FRAMES**    **NO FRAMES**     **All Classes** |  |
| SUMMARY: NESTED \| ENUM CONSTANTS \| FIELD \| METHOD | DETAIL: ENUM CONSTANTS \| FIELD \| METHOD |  |


---

# *Copyright © 2007-2008*
